# Integrated gut metagenomic and muscle proteomic analysis reveals the role of dietary fermented extruded brewers’ spent grain in enhancing pork quality through the gut-muscle axis

**DOI:** 10.1186/s40104-026-01429-4

**Published:** 2026-06-06

**Authors:** Yang Liu, Yueqin Xie, Jianqi Yang, Yu Deng, Dongyun Liu, Junlei Chang, Jiayong Tang, Hua Zhao, Xiaoling Chen, Gang Tian, Guangmang Liu, Jingyi Cai, Gang Jia

**Affiliations:** Key Laboratory for Animal Disease-Resistance Nutrition of China, Ministry of Education, Institute of Animal Nutrition, Sichuan Agricultural University, No. 211 Huimin Road, Chengdu, Sichuan 611130 China

**Keywords:** Fermented extruded brewers’ spent grain, Growth performance, Gut-muscle axis, Growing-finishing pigs, Meat quality

## Abstract

**Background:**

The fact that feeding pigs with probiotic-fermented agricultural by-products improves pork quality has been repeatedly demonstrated and widely applied, but the underlying mechanisms remain unclear. This study explored the effects of fermented extruded brewers’ spent grain (FEBSG) on meat quality in growing-finishing pigs, as well as its regulatory mechanisms.

**Methods:**

Sixty Duroc × Landrace × Yorkshire pigs (52.25 ± 2.10 kg) were randomly assigned to five dietary treatments, in which FEBSG replaced 0, 5%, 10%, 15%, and 20% of soybean meal (SBM). The experiment spanned 10 weeks.

**Results:**

Compared with the control, 20% FEBSG significantly increased final body weight, average daily feed intake, and average daily gain, while decreasing feed to gain ratio (*P* < 0.05). Both 15% and 20% FEBSG improved carcass characteristics and meat quality, including higher carcass weight, loin eye area, and intramuscular fat content, along with lower drip loss and shear force (*P* < 0.05). These treatments also enhanced flavor-related amino acids and unsaturated fatty acids (*P* < 0.05), and improved umami and sweet taste profiles. Moreover, 20% FEBSG increased muscle fiber density and reduced fiber diameter, upregulated *MyHC I*, *MyHC IIa*, *PGC-1α*, *AMPKα1*, *TFAM*, and SDH activity, and downregulated *MyHC IIb* and LDH activity (*P* < 0.05). Proteomic analysis identified 69 differentially expressed proteins, with enrichment in AMPK and PPAR signaling pathways. Metagenomic analysis revealed increased abundance of short-chain fatty acid-producing bacteria, including *Clostridium*, *Lactobacillus*, *Prevotella*, and *Bartonella*. Correlation analysis demonstrated associations between gut microbiota diversity and meat quality traits, as well as between dominant microbial genera and differentially expressed proteins, volatile fatty acids, muscle fiber characteristics, and the AMPK/PGC-1α/TFAM signaling pathway.

**Conclusions:**

Partial replacement of SBM with FEBSG positively influenced growth performance and pork quality in pigs, with the underlying mechanisms may involve the activation of the AMPK/PGC-1α/TFAM signaling pathway via the gut-muscle axis, thereby enhancing mitochondrial biogenesis, muscle development, and metabolism.

**Supplementary Information:**

The online version contains supplementary material available at 10.1186/s40104-026-01429-4.

## Background

Brewers’ spent grain (BSG), a main byproduct of beer manufacturing, is generated through the fermentation and extraction of soluble carbohydrates from cereals, followed by pressing, filtration, and drying [1]. In China, the annual production of BSG is approximately 2 million tons, making it a cost-effective feed ingredient due to its richness in nutrients essential for animal diets [2]. It has been reported that transforming BSG waste into animal feed via sustainable methods can facilitate a shift from a linear to a circular economy, reducing pressure on food resources and fostering environmental conservation [3]. However, major challenges in utilizing BSG as animal feed include its high moisture content and resistance to degradation due to high fiber levels, which lead to nutrient loss and reduced absorption efficiency, thereby limiting its large-scale application [4]. To overcome these challenges, our latest research has shown that the combination of extrusion pretreatment and probiotic solid-state fermentation can disrupt the anti-degradation barrier structure of BSG, improve its digestibility and nutritional value, and thereby effectively address the above challenges [5, 6].

Pork is widely consumed because of its high nutritional value, delicious taste, and significant protein content. Economic growth and improved living standards have resulted in a growing demand for premium pork in recent years [7]. This trend underscores the importance of enhancing pork quality to promote the development of sustainable animal husbandry. The quality of pork is defined by complex traits, including sensory quality, meat color, nutritional value, pH value, intramuscular fat, and flavor substance content [8, 9]. Consumers typically base their meat purchasing decisions on visual perception such as meat color, freshness, lean percentage, and the visible drip content, whereas the qualities they focus on when eating meat are taste, tenderness, and juiciness [10]. These sensory aspects of pork quality are affected by factors including pH value, water retention properties, shear force, and the color of the meat [11]. However, the rapid expansion of intensive farming and the focus on the pursuit of productivity and lean meat output, has negatively impacted the quality of pork. This presents significant challenges for producing high-quality pork. Consequently, improving the quality and flavor of pork is crucial to meet consumer needs and ensure the production of superior pork.

Nutritional modulation, particularly through the use of fermented feed, has emerged as an effective strategy to improve both animal performance and product quality [12]. Fermented feeds containing probiotics and their bioactive metabolites have been widely used in livestock production, offering benefits such as enhanced growth performance, immune function, nutrient absorption, and feed efficiency [13]. Furthermore, the widespread application of fermented feed contributes to alleviating the strain on feed resources, increasing the utilization of agricultural byproducts, and addressing bottlenecks in the feed and livestock industries [14]. Recent studies indicate that fermenting agricultural residues like BSG with probiotics has shown positive effects on pork quality traits such as meat color, tenderness, taste, and juiciness [15–17]. However, the regulatory mechanisms underlying these observation remains unknown.

In recent years, the interaction between gut microbiota and host skeletal muscle (known as the gut muscle axis) has attracted increasing attention, especially in the context of human health [18]. Emerging evidence suggests that the intestinal microbiota can influence muscle physiology and function through the production of bioactive metabolites such as short-chain fatty acids, amino acids, and microbial peptides [19]. These metabolites are capable of modulating host metabolism, inflammation, mitochondrial function, and muscle fiber composition. Animal studies have begun to elucidate similar mechanisms. Dou et al. [20] demonstrated that supplementation with *Clostridium butyricum* altered the gut microbiota composition and significantly improved skeletal muscle development and meat quality in lambs by modulating the gut–muscle axis. Similarly, Xu et al. [21] reported that the gut microbiota shaped by dietary lactic acid bacteria influenced the flavor profile of duck meat, highlighting the role of microbial-derived metabolites in regulating meat quality. Therefore, the aim of this study was to evaluate the effects of dietary fermented extruded brewers’ spent grain (FEBSG) on pork quality in growing-finishing pigs and to elucidate the underlying mechanisms. By integrating gut metagenomics and muscle proteomics, we aimed to explore how FEBSG modulates the gut–muscle axis and activates the AMPK/PGC-1α/TFAM signaling pathway, contributing to improvements in meat quality. Additionally, considering the limited availability of soybean meal (SBM) and the growing competition for food resources between humans and animals, this study proposes a sustainable feed strategy that reduces reliance on SBM while enhancing pork quality through dietary microbiota modulation.

## Materials and methods

### Preparation of fermented extruded brewers’ spent grain

The extruded BSG was sourced from Sichuan Nuke Teide Biotechnology Co., Ltd., located in Chengdu, China. The pilot-scale production of FEBSG took place at the Animal Experiment base of Sichuan Agricultural University. FEBSG was prepared using probiotics including *Bacillus safensis* SCYA3, *Candida tropicalis* SCYA4, and *Bacillus subtilis* SCYA6 (kept in the laboratory) according to a previously optimized fermentation method. The basal substrate contained 90% extruded brewers’ spent grain and 10% wheat bran. The fermentation conditions as follows: a blend of *Bacillus safensis* SCYA3, *Bacillus subtilis* SCYA6, and *Candida tropicalis* SCYA4 in a ratio of 3:2:1, an inoculation volume of 15% (v/w), and a substrate to water ratio of 1:1 (w/v). Subsequently, the combined substrate was placed into a plastic bag that had a one-way valve fitted, sealed, and allowed to ferment at 34 °C for 7 d. In our previous study, we displayed the nutritional level of FEBSG before and after fermentation [6].

### Experimental animals and feeding

Sixty healthy crossbred pigs (Duroc × Landrace × Yorkshire), all 110 days old and averaging 52.25 ± 2.09 kg in weight (half male and half female), were assigned at random to five treatment: Con group, 5%FEBSG group (replacing 5% SBM with FEBSG), 10%FEBSG group (replacing 10% SBM with FEBSG), 15%FEBSG group (replacing 15% SBM with FEBSG), 20%FEBSG group (replacing 20% SBM with FEBSG). Each treatment included six replicates, each with two pigs. The basal diet (Con group) was designed following the NRC (2012) guidelines for weight ranges of 50 to 75 kg, 75 to 100 kg, and 100 to 125 kg [22]. Table 1 presented the nutritional content and feed composition of the basal diet. Pigs had drink water freely and were fed daily at 8:00, 14:00, and 20:00. The experiment spanned 10 weeks. The animal feeding and management procedures for this research were endorsed by the Animal Ethics Committee at Sichuan Agricultural University (No: YYS20190612). Daily records of the pigs’ feed consumption were maintained, and their body weights were determined at 8:00 on d 1 and 70.

**Table 1:** Ingredient and nutrient composition of the basal diet (air-dry basis)

Ingredients, %	Phase, kg		
	50–75	75–100	100–125
Corn	75.51	80.88	84.48
Soybean meal	20.00	15.00	11.44
Soybean oil	1.55	1.46	1.40
L-Lysine HCl	0.31	0.30	0.38
DL-Methionine	0.08	0.06	0.05
Threonine	0.09	0.09	0.10
Tryptophan	0.02	0.02	0.03
Choline chlorine	0.10	0.10	0.10
Limestone	0.67	0.70	0.73
CaHPO_4_	1.15	0.86	0.76
NaCl	0.30	0.30	0.30
Mineral premix^1^	0.20	0.20	0.20
Vitamin premix^2^	0.02	0.03	0.03
Total	100.00	100.00	100.00
Nutrients levels			
Digestible energy, MJ/kg^3^	14.08	14.10	14.09
Crude protein^4^	14.83	13.12	11.80
Calcium^4^	0.58	0.53	0.47
Total Phosphorus^4^	0.53	0.47	0.42

^1^Mineral premix provides per kg of basal diet: 50–75 kg: Fe, 50.0 mg, Cu, 4.0 mg, Mn, 2.0 mg; Zn, 60.0 mg, I, 0.14 mg, Se, 0.15 mg. 75–125 kg: Fe, 40.0 mg, Cu, 3.0 mg, Mn, 20.0 mg; Zn, 50.0 mg, I, 0.14 mg, Se, 0.15 mg

^2^Vitamin premix provides per kg of basal diet: Vitamin A, 9,000 IU; Vitamin D_3_, 3,000 IU; Vitamin E, 20.0 IU; Vitamin K_3_, 3.0 mg; Vitamin B_1_, 1.5 mg; Vitamin B_2_, 4.0 mg; Vitamin B_6_, 3.0 mg; Vitamin B_12_, 0.02 mg; Nicotinamide, 30 mg; Pantothenic, 15.0 mg; Folic acid, 0.75 mg; Biotin, 0.1 mg

^3^Digestible energy was calculated based on database of NRC (2012) [22]

^4^Values determined by analysis; each value is based on triplicate determinations

### Sample collection

The pigs underwent a 24-h fast prior to being weighed and subsequently slaughtered at the end of the experiment. 30 pigs (6 pigs per group) were transferred to a commercial abattoir (25 min away), and slaughtered by electrical stunning, exsanguinated, and eviscerated according to standard procedures. Following slaughter, individual carcass weights were taken and documented to calculate the dressing percentage. According to the requirements of NY/T 825–2004 [23], backfat thickness was gauged at three distinct sites using a vernier caliper: the first rib, the last rib, and the last lumbar vertebra. Furthermore, loin eye area was determined at the last rib using planimetry. Subsequently, colon digesta samples were collected using sterilized equipment and preserved at −80 °C for analyzing intestinal microorganisms and volatile fatty acids. Additionally, within 15 min after slaughter, the left side’s longissimus thoracis (LT) was sectioned into three portions. The first part was promptly collected and stored in liquid nitrogen for future analysis. The second part was immersed in 4% paraformaldehyde to examine muscle fiber morphology. The third part was designated for assessing shear force, cooking loss, pH, meat color, drip loss, and marbling score. It was then kept in a refrigerated environment at 4 °C for 24 h to assess the pork quality.

### Meat quality analysis

The pH of the LT was measured 45 min and 24 h post-slaughter, utilizing a portable pH meter. Additionally, 45 min post-mortem, the meat color parameters including lightness (L*), redness (a*), and yellowness (b*) were assessed using a CR-410 chromometer (Konica Minolta, Tokyo, Japan, Illuminant D65, observer angle of 10^◦^ with zero, aperture size of 5.0 mm) after 1 h of blooming. Around 30 g of LT sample was initially weighed 45 min postmortem to assess drip loss. Following this, the sample was sealed in a bag and refrigerated at 4 °C, after which it was reweighed after a period of 24 h. Shear force and cooking loss: samples after 24 h of post-mortem aging (approx. 80 g) were individually placed in polythene bags and cooked in a same water bath (80 °C). When the internal temperature reached 70 °C, sample was removed and cooled to room temperature. It should be pointed that there was only one cooking batch and one sample was prepared especially for the determination of the central temperature. Sear force: the cooked samples were cut into rectangle (cross-sectional area 1 cm^2^), a tenderness meter (Sichuan Agricultural University, Chengdu, China) equipped with a strain gauge load cell (capacity of 50 kg) was used to assess shear force, each sample was assayed > 8 times. The marbling score was assessed 45 min postmortem, using the NPPC meat color chart as a reference guide. Following this, roughly 50 g of the LT was initially weighed and placed into a container that had been pre-weighed. The sample was subsequently freeze-dried at −80 °C for 48 h. Once the freeze-drying was complete, the container's weight was measured again to ascertain the LT’s moisture content by calculating the weight difference. The dried muscle tissues were determined for their crude protein, intramuscular fat, amino acids, and fatty acids content.

### Electronic tongue analysis

The taste assessment of the LT was conducted with an electronic tongue system (Alpha MOS Company, Toulouse, France). Specifically, samples weighing 10 g were homogenized for 1 min in appropriate amount of deionized water. After 30 min of standing, the 80 mL supernatants were obtained through centrifugation and filtration for electronic tongue analysis.

### H&E staining

Following a 24-h fixation period, the LT samples were dehydrated and embedded in preparation for sectioning. Samples were sectioned to a thickness of 10 μm using an ultramicrotome. The paraffin-embedded sections were then subjected to dewaxing and rehydration. Afterwards, they were stained according to the protocol specified in the H&E staining kit. Ultimately, the fiber density and muscle fiber diameter were determined using Image-Pro Plus 6.0 software.

### Metabolic enzyme activity assay

A sample of the LT sample, approximately 0.1 g, was homogenized on ice with 900 μL of 0.9% saline solution. After homogenization, the supernatant was obtained by centrifuging at 2,500 × *g* for 10 min at 4 °C. Then, the activity of lactate dehydrogenase (LDH), malate dehydrogenase (MDH), and succinic dehydrogenase (SDH) were measured using commercial assay kits following the supplier’s protocol (Nanjing Jiancheng Bioengineering Institute, Nanjing, China).

### Real-time quantitative PCR

Total RNA was extracted from LT (30 mg) using RNAiso Plus reagent following the manufacturer’s protocol. Subsequently, the RNA concentration was quantified. Then, total RNA was reverse transcribed into cDNA following the provided protocol. Real‑time quantitative PCR was performed using the 7900HT real-time PCR system in accordance with the established procedure. Primer sequences were detailed in Table S1. The mRNA expression was calculated using comparative method (2^−ΔΔCT^). GAPDH served as the internal control.

### Immunofluorescence staining

The LT sample was fixed with a 4% paraformaldehyde solution, and then cut into 10-µm sections with a microtome. The sections were treated with primary antibodies against slow myosin heavy chain (MyHC) and fast MyHC (Abcam) for an overnight period. Afterward, they were incubated with secondary antibodies: CY3-conjugated goat anti-rabbit IgG and Alexa Fluor 488-conjugated goat anti-mouse IgG. Then the sections were processed with an autofluorescence suppression reagent and counterstained with DAPI for nuclear visualization in a dark environment. Ultimately, images for analysis were captured using fluorescence microscope to quantify the proportion of slow and fast muscle fibers.

### Proteomics analysis

#### Protein extraction and labelling

Protein from the LT was extracted with a lysis buffer, then the protein was digested with trypsin and ammonium bicarbonate buffer for 4 h. Following this, trypsin and CaCl_2_ were added to the samples, which were left overnight. The next day, the supernatant was collected and washed, then the filtrate was dissolved in TEBA buffer. Subsequently, the mixture was supplemented with TMT-tagged reagents. Finally, the reaction was stopped using ammonium hydroxide.

#### Separation of fractions and LC–MS/MS analysis

A gradient elution procedure utilizing acetonitrile concentrations of 2% and 98% was performed to separate components within the sample. This process was conducted on an L3000 HPLC system from Rigol. Post-fractionation, the resulting sample fractions were subjected to lyophilization to remove solvents, followed by resuspension in a 0.1% formic acid solution to prepare them for further analysis. The subsequent analytical steps were executed on the EASY-nLC™ 1200 UPLC system.

#### Data analysis

Peptide identification and quantification against the UniProt Proteomes-Sus scrofa database were performed using MaxQuant software. The criteria for peptide and protein identification were set with a maximum false discovery rate (FDR) of 1%. Proteins were considered up-regulated if they had a fold change > 1.2 and a *P* < 0.05. Proteins were identified as down-regulated with a fold change < 0.83 and a *P* < 0.05. GO enrichment and KEGG pathway analyses were conducted using the DAVID database. Following this, interaction networks of the differentially expressed proteins were built using the STRING database.

### Metagenomics analysis

DNA extracted from colon digesta samples was evaluated for quality through gel electrophoresis. It was then fragmented with the dsDNA Fragmentase enzyme (NEB, M0348S). Subsequently, the paired-end library was constructed using the TruSeq Nano DNA LT Library Preparation Kit (FC-121-4001), adhering strictly to the manufacturer’s protocol. This involved the precise steps of DNA fragment ligation, purification, and enrichment. The library was subsequently analyzed for size distribution and quantified using qPCR, proceeding to PE150 sequencing. The raw sequencing reads were meticulously processed for quality control with Fastqc (version 0.10.0), which facilitated the trimming of adapter sequences and the removal of substandard reads. The high-quality reads that passed the filtration were then assembled in unison using the Megahit software (version 1.2.9). The assembled sequences were subjected to gene prediction using MetaGeneMark (version 3.2.6). An annotation of microbial functions was done using the KEGG (version 87.1) and CAZymes (version 8) databases.

### Determination of short-chain fatty acids

Approximately 0.5 g of colon digesta were homogenized in 2 mL of ultrapure water, followed by centrifugation at 3,000 × *g* for 15 min. The supernatant was then combined with an ice-cold metaphosphoric acid solution and kept at 4 °C for 30 min before another centrifugation. The levels of acetic acid (AA), propionic acid (PA), isobutyric acid (IBA), butyric acid (BA), valeric acid (VA), and isovaleric acid (IVA) were subsequently analyzed using a GC 8890 series gas chromatograph (Agilent Technologies, Santa Clara, CA, USA).

### Statistical analysis

The performance characteristics data were analyzed using a one-way analysis of variance (ANOVA) followed by Duncan’s multiple range test. SPSS 22.0 software (Chicago, IL, USA) was used for the analysis. Results were displayed as the means and standard error of the mean (SEM). The regression analysis was employed to test the linear and quadratic effects. *P* < 0.05 indicated statistically significant differences.

## Results

### Growth performance and carcass properties

The production performance and carcass characteristics of the pigs were shown in Table 2. Pigs fed 15% and 20% FEBSG diets exhibited significantly higher (*P* < 0.05) final body weight, average daily feed intake, carcass weight and loin-eye area compared to those fed Con diets. Moreover, the 20% FEBSG group displayed a significant increase in average daily gain and a significant decrease in feed to gain ratio compared to those fed Con diets (*P* < 0.05). While no significant differences were observed in dressing percentage, carcass length and backfat thickness in any groups (*P* > 0.05). Interestingly, the final body weight, average daily feed intake, average daily gain, feed to gain ratio, carcass weight and loin-eye area of pigs showed linear and quadratic relationships with FEBSG (*P* < 0.05).

**Table 2:** Effects of different diet FEBSG treatment on growth performance and carcass properties in growing-finishing pigs

Items	Control	5%FEBSG	10%FEBSG	15%FEBSG	20%FEBSG	SEM	*P*-value		
							ANOVA	Linear	Quadratic
Growth performance									
Initial body weight, kg	52.29	52.25	52.21	52.25	52.25	0.38	1.000	0.976	0.998
Final body weight, kg	118.17^b^	117.66^b^	123.54^a^	125.29^a^	127.42^a^	0.96	< 0.01	< 0.01	< 0.01
Average daily feed intake, kg/d	2.84^b^	2.85^ab^	3.02^ab^	3.05^ab^	3.11^a^	0.04	0.07	0.004	0.018
Average daily gain, kg/d	0.94^b^	0.94^b^	1.02^a^	1.04^a^	1.08^a^	0.01	0.001	< 0.01	< 0.01
Feed to gain ratio	3.02^ab^	3.05^a^	2.97^abc^	2.92^bc^	2.89^c^	0.02	0.037	0.003	0.010
Carcass properties									
Carcass weight, kg	83.47^c^	83.93^c^	85.10^bc^	89.67^ab^	92.77^a^	1.01	0.004	< 0.01	< 0.01
Dressing percentage, %	72.99	71.52	70.98	72.92	73.54	0.77	0.831	0.653	0.541
Carcass length, cm	100.83	102.28	104.92	103.55	105.17	0.76	0.338	0.062	0.151
Backfat thickness, cm	2.74	2.98	2.89	2.80	2.86	0.07	0.873	0.905	0.838
Loin-eye area, cm^2^	44.04^b^	46.58^ab^	47.80^a^	49.62^a^	50.13^a^	0.64	0.010	< 0.01	0.001

Means with different superscripts in the same row indicate significantly difference (*P* < 0.05; *n* = 6)

### Meat quality

Table 3 showed significant differences between the Con group and those fed 10%, 15%, and 20% FEBSG diets. Specifically, 15% and 20% FEBSG groups exhibited significantly (*P* < 0.05) higher value of a* (redness) and intramuscular fat, and significantly lower value of drip loss compared to those fed Con diets. Furthermore, the 20% FEBSG groups exhibited lower cooking loss and shear force, while exhibited higher marbling scores than those on the Con diet. The 15% FEBSG group exhibited significantly (*P* < 0.05) higher value of pH_24h_. While no significant variations (*P* > 0.05) were discovered in pH_45min_, L* (lightness), b* (yellowness), crude protein and moisture content among pigs fed 0%, 5%, 10%, 15%, and 20% FEBSG diets. While pH_24h_, a*, drip loss, cooking loss, shear force, and intramuscular fat of pigs showed linear and quadratic relationships with FEBSG (*P* < 0.05).

**Table 3:** Effects of different diet FEBSG treatment on meat quality in growing-finishing pigs

Items	Control	5%FEBSG	10%FEBSG	15%FEBSG	20%FEBSG	SEM	*P*-value		
							ANOVA	Linear	Quadratic
pH_45min_	6.34	6.29	6.22	6.31	6.27	0.03	0.843	0.565	0.692
pH_24h_	5.37^b^	5.40^ab^	5.44^ab^	5.45^a^	5.44^ab^	0.01	0.118	0.013	0.026
L*_45min_	41.65	42.78	42.81	40.95	41.22	0.28	0.097	0.183	0.125
a*_45min_	7.71^b^	8.05^ab^	8.66^ab^	9.05^a^	8.98^a^	0.19	0.086	0.006	0.018
b*_45min_	10.59	10.94	10.82	11.08	10.33	0.11	0.208	0.620	0.118
Drip loss, %	2.71^a^	2.42^ab^	2.19^b^	2.08^b^	2.03^b^	0.08	0.040	0.002	0.005
Cooking loss, %	33.54^a^	32.25^a^	30.97^ab^	31.25^ab^	29.19^b^	0.46	0.030	0.002	0.007
Shear forces, N	57.74^ab^	59.46^a^	52.61^bc^	53.73^abc^	49.59^c^	1.05	0.011	0.003	0.006
Marbling score	2.17^b^	2.50^ab^	2.67^ab^	2.83^ab^	3.17^a^	0.14	0.215	0.014	0.053
Moisture, %	70.71	71.12	72.85	72.43	71.98	0.49	0.635	0.269	0.348
Intramuscular fat, %	2.37^c^	2.52^bc^	2.80^ab^	3.01^a^	3.06^a^	0.07	0.003	< 0.01	< 0.01
Crude protein, %	21.14	20.72	23.14	22.94	24.24	0.59	0.297	0.040	0.126

Means with different superscripts in the same row indicate significantly difference (*P* < 0.05; *n* = 6)

### Amino acid, taste characteristics and fatty acid profiles

**Fig. 1:**
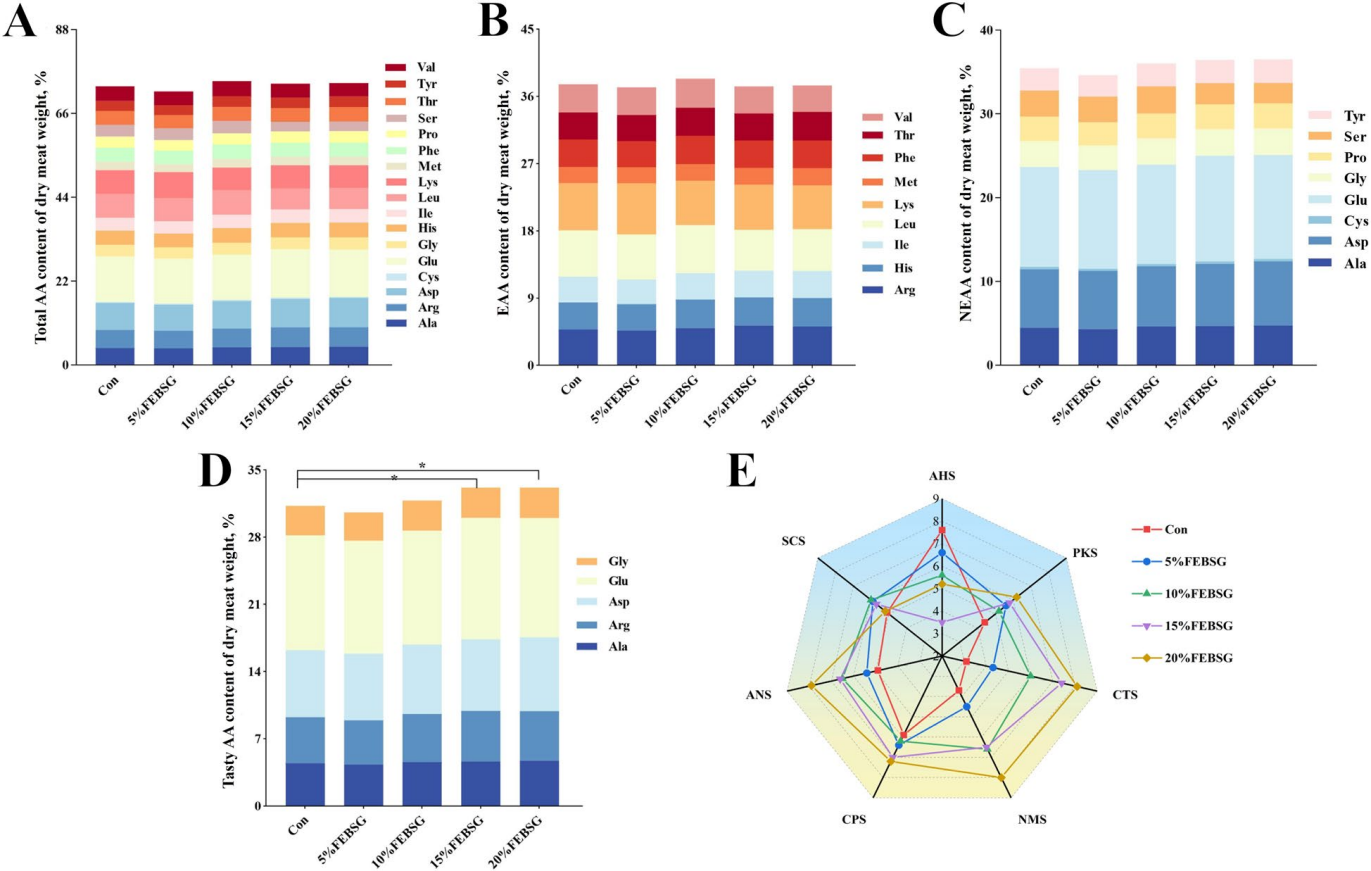
Effects of different diet FEBSG treatment on amino acid and taste characteristics in growing-finishing pigs. **A** Total amino acids. **B** Essential amino acids. **C** Non-essential amino acids. **D** Tasty amino acids. **E** E-tongue radar plot. The results were presented as mean ± SEM (*n* = 6). Bars with different letters indicates significant difference (*P* < 0.05)

The composition of amino acids (AA) in the LT were analyzed, revealing that no significant differences were observed in total AA, non-essential AA (NEAA) and essential AA (EAA) in any groups (*P* > 0.05) (Fig. 1A–C), while pigs fed diets containing 15% and 20% FEBSG had significantly higher (*P* < 0.05) contents of tasty AA compared to pigs fed Con diets (Fig. 1D). Additionally, the taste characteristics of pork meat were assessed using an electronic tongue (e-tongue) across different groups. As shown in Fig. 1E, significant variations were displayed in the intensity values of the AHS, ANS, and NMS sensors among the five groups. The 15% and 20% FEBSG groups exhibited higher intensity values for the ANS and NMS sensors than the Con group. Conversely, these groups showed significantly lower (*P* < 0.05) intensity values for the AHS sensors than the Con group. Additionally, the fatty acid profiles in the LT were presented in Table 4. The levels of oleic acid (C18:1n9c), MUFA, PUFA, and n-6 PUFA were significantly higher (*P* < 0.05) in the 15% and 20% FEBSG groups compared to the Con group. Additionally, the PUFA/SFA ratio was higher in the 15% FEBSG group than in the Con group. However, the dietary treatments did not have a significant effect on the levels of SFA, n-3 PUFA, and the n-6/n-3 ratio (*P* > 0.05). While the content of C18:1n9c, C18:2n6c, MUFA, PUFA, n-6 PUFA and PUFA/SFA of pigs showed linear and quadratic relationships with FEBSG (*P* < 0.05).

**Table 4:** Effects of different diet FEBSG treatment on fatty acid composition in growing-finishing pigs, % of dry meat weight

Items	Control	5%FEBSG	10%FEBSG	15%FEBSG	20%FEBSG	SEM	*P*-value		
							ANOVA	Linear	Quadratic
C10:0	0.97	0.89	0.41	0.37	0.73	0.13	0.512	0.298	0.285
C14:0	1.39^ab^	1.49^a^	1.45^ab^	1.33^b^	1.46^ab^	0.02	0.193	0.887	0.988
C16:0	23.56^ab^	24.48^ab^	24.98^a^	23.16^b^	25.03^a^	0.24	0.033	0.357	0.646
C16:1	3.07	3.29	3.39	3.64	3.46	0.09	0.400	0.081	0.240
C17:0	0.36	0.27	0.30	0.36	0.32	0.01	0.238	0.861	0.559
C18:0	10.61	11.19	11.04	9.97	10.39	0.19	0.242	0.219	0.350
C18:1n9c	41.01^c^	42.37^bc^	40.88^c^	44.17^b^	46.78^a^	0.52	< 0.01	< 0.01	< 0.01
C18:2n6c	13.93^b^	12.69^b^	14.11^b^	17.90^a^	16.90^a^	0.50	0.001	0.001	0.003
C20:0	0.15	0.16	0.17	0.15	0.15	0.01	0.282	0.776	0.462
C18:3n6	0.09^ab^	0.07^b^	0.09^ab^	0.12^a^	0.11^a^	0.01	0.011	0.010	0.024
C18:3n3	0.80^ab^	0.75^ab^	0.75^b^	0.89^a^	0.80^ab^	0.02	0.768	0.340	0.580
C20:2	0.55	0.49	0.51	0.61	0.56	0.02	0.226	0.321	0.488
C20:3n6	0.25^ab^	0.20^b^	0.26^ab^	0.32^a^	0.29^a^	0.01	0.075	0.045	0.129
C20:3n3	0.11	0.10	0.11	0.13	0.12	0.01	0.194	0.128	0.292
SFA	37.02^ab^	38.48^a^	38.36^a^	35.33^b^	38.09^a^	0.42	0.079	0.736	0.942
MUFA	44.12^c^	45.71^bc^	44.33^c^	47.86^ab^	50.30^a^	0.56	< 0.01	< 0.01	< 0.01
PUFA	15.93^b^	14.44^b^	16.04^b^	20.20^a^	19.05^a^	0.55	0.001	0.001	0.004
n-3 PUFA	1.11	0.99	1.06	1.27	1.19	0.03	0.059	0.061	0.131
n-6 PUFA	14.28^b^	12.96^b^	14.46^b^	18.33^a^	17.31^a^	0.52	0.001	0.001	0.003
PUFA/SFA	0.43^bc^	0.38^c^	0.42^bc^	0.58^a^	0.50^ab^	0.02	0.001	0.005	0.017
n-6/n-3	13.56	13.03	13.67	14.50	14.51	0.33	0.594	0.157	0.336

Means with different superscripts in the same row indicate significantly difference (*P* < 0.05; *n* = 6). *SFA* Saturated fatty acid, *MUFA* Monounsaturated fatty acid, *PUFA* Polyunsaturated fatty acid, *n-6 PUFA* Omega 6 polyunsaturated fatty acid, *n-3 PUFA* Total mega 3 polyunsaturated fatty acid

### Muscle fiber characteristic and muscle fiber type transformations

**Fig. 2:**
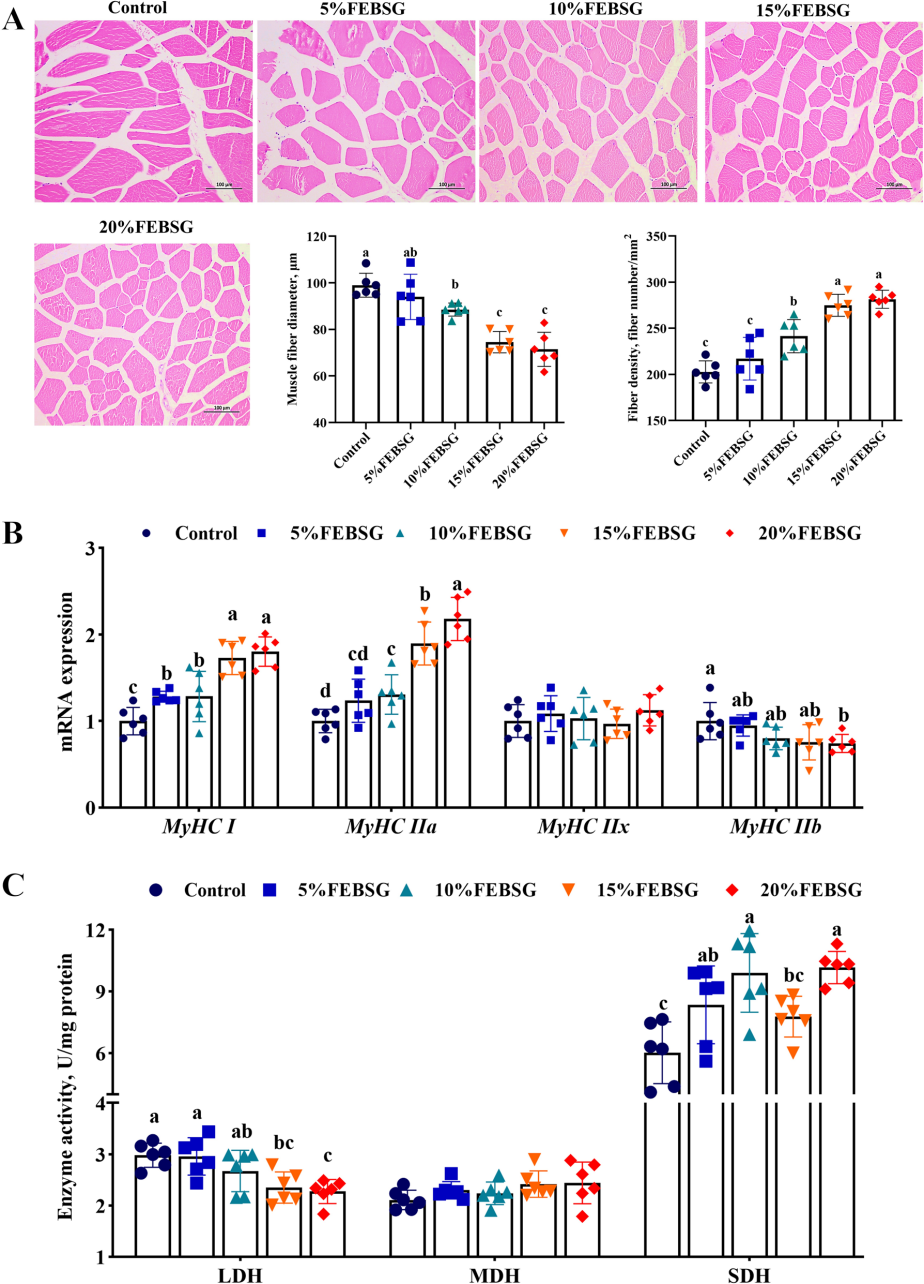
Effects of different diet FEBSG treatment on muscle fiber characteristics in growing-finishing pigs. **A** Graph of HE staining, muscle fiber diameter and fiber density of LT. **B** The mRNA levels of genes related to muscle fiber-type transformation. **C** Activities of LDH, MDH and SDH. Data are expressed as mean ± SEM (*n* = 6), and different letters denote significant differences (*P* < 0.05)

**Fig. 3:**
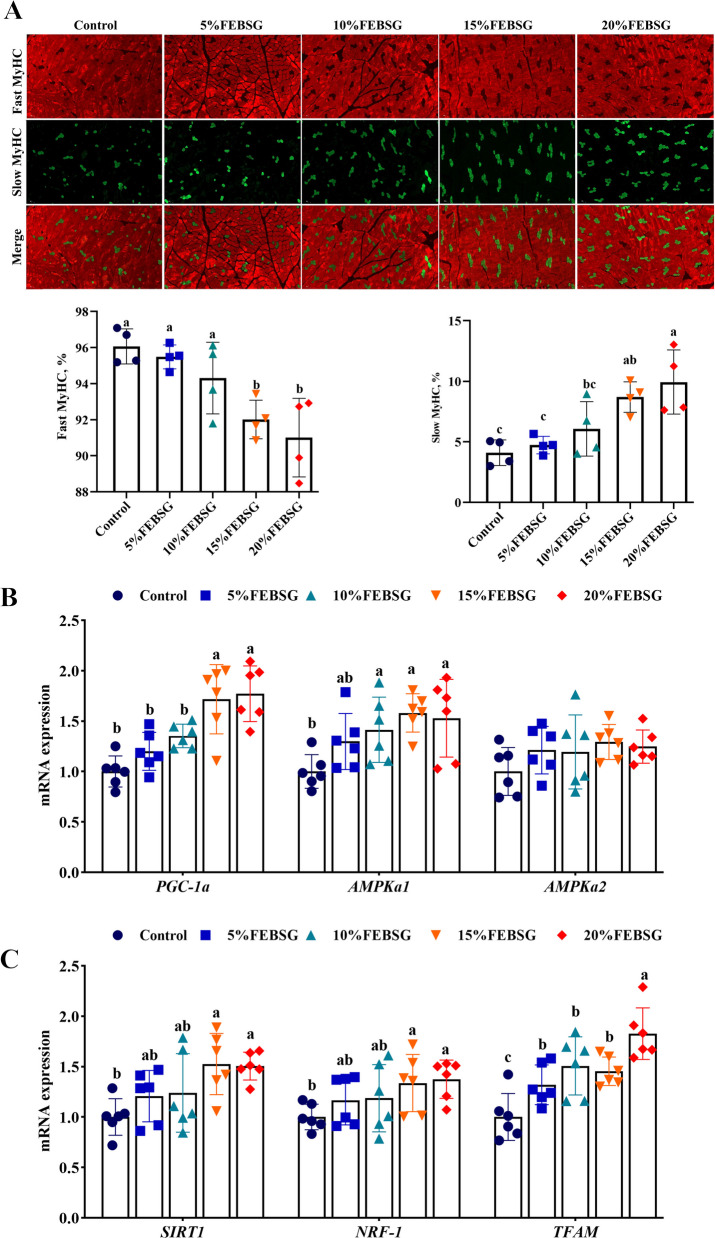
Effects of different diet FEBSG treatment on muscle fiber type transformations and mitochondrial function in growing-finishing pigs. **A** Immunofluorescent staining of slow MyHC and fast MyHC in the LT (red is fast-twitch fiber and green is slow-twitch fiber), percentage of the fast myhc and percentage of the slow MyHC in the LT. **B** and **C** Gene expression associated with AMPK signaling and mitochondrial biogenesis regulators. Data are expressed as mean ± SEM (*n* = 6), and different letters denote significant differences (*P* < 0.05)

The muscle fiber diameter significantly decreased, while the fiber density markedly (*P* < 0.05) increased in the 10%, 15%, and 20% FEBSG groups, as depicted in Fig. 2A. The mRNA levels of genes related to muscle fiber-type transformation were also evaluated. The 10%, 15%, and 20% FEBSG groups markedly increased (*P* < 0.05) the gene expression levels of *MyHC I* and *MyHC IIa*, while 20% FEBSG group significantly decreased (*P* < 0.05) the mRNA level of *MyHC IIb* when compared to the Con group. Nevertheless, no significant variation (*P* > 0.05) was observed in the mRNA level of *MyHC IIx* in any groups (Fig. 2B). The metabolic enzyme activities in the LT were also assessed. The 15% and 20% FEBSG groups significantly decreased LDH activity while 5%, 10% and 20% FEBSG groups significantly increasing SDH activities (*P* < 0.05), and no significant variation (*P* > 0.05) was observed in the MDH activity in any groups (Fig. 2C). Furthermore, immunofluorescence results indicated that the 15% and 20% FEBSG groups significantly decreased the percentage of fast MyHC and enhanced the percentage of slow MyHC (*P* < 0.05) (Fig. 3A).

### Gene expression associated with AMPK signaling and mitochondrial biogenesis regulators

The effects of FEBSG on the AMPK signaling pathway and regulators of mitochondrial biogenesis in finishing pigs were illustrated in Fig. 3B and C. The 15% and 20% FEBSG groups had significantly increased (*P* < 0.05) expression of *PGC-1α*, *AMPKα1*, *SIRT1*, *TFAM* and *NRF-1* compared to the Con group. Nevertheless, no significant variation (*P* > 0.05) was observed in the mRNA level of *AMPKα2* in any groups.

### Proteomics analysis of the LT

**Fig. 4:**
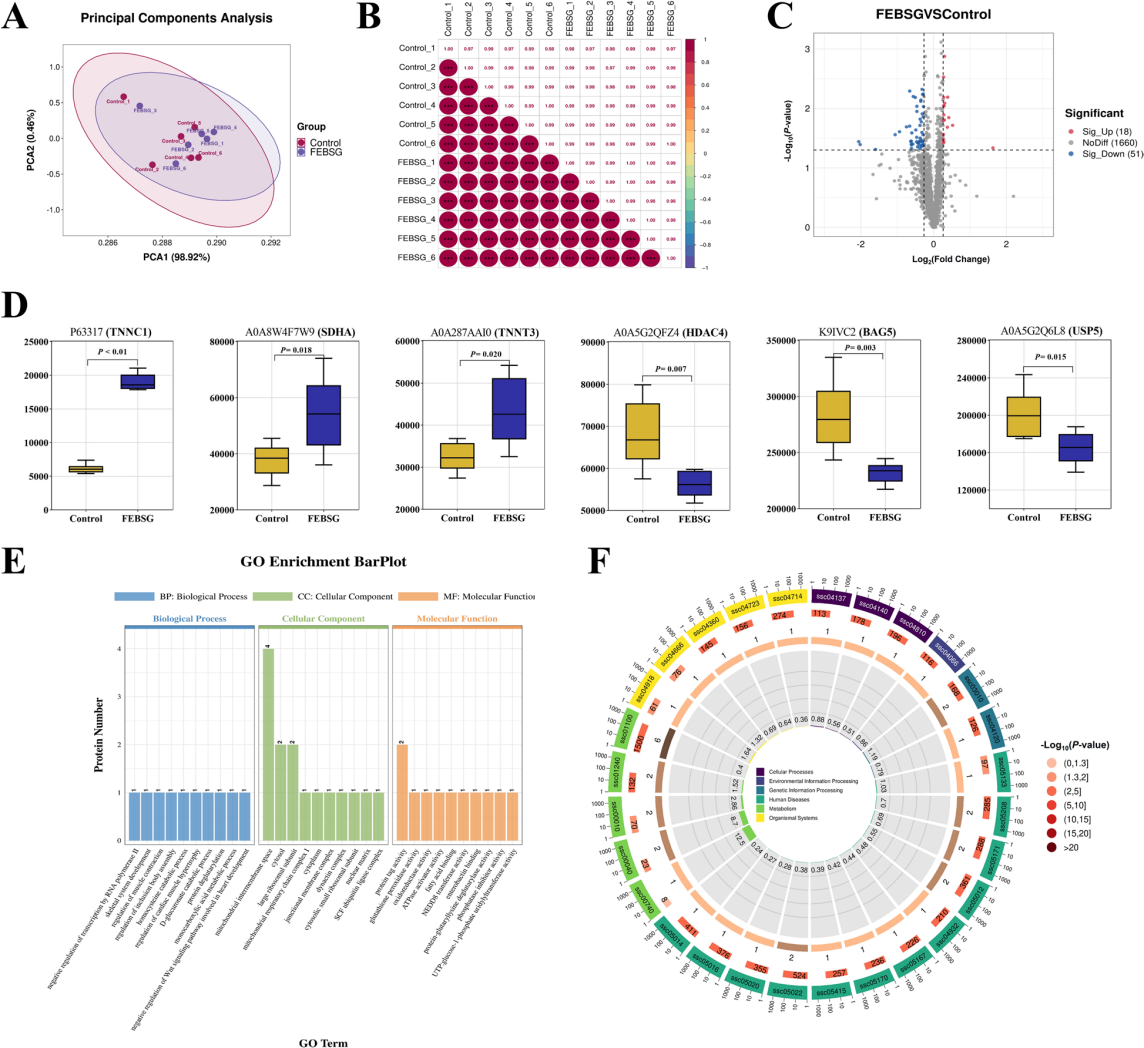
TMT-based quantitative proteomic analysis of the LT. **A** Principal component analysis (PCA) score plot of proteomic data. **B** Sample correlation analysis. On the upper right of the diagonal line of the image, the number indicates the correlation value of the two samples, * indicates the degree of significance (^*^P < 0.05, ^**^P < 0.01, ^***^P < 0.001). **C** Volcano plot of differentially expressed proteins between the control and 20% FEBSG. The proteins up- or down-regulated are indicated in red and blue, respectively. **D** Box plot of the differential protein expression levels of the top 3 (up and down, respectively) with the biggest fold change values. **E** Gene Ontology (GO) annotation plot of differentially expressed proteins. BP, Biological Process; MF, Molecular Function; CC, Cellular Component. **F** KEGG pathway enrichment analysis

Further assessment of muscle fiber traits in finishing pigs was conducted through quantitative proteomic analysis of LT using TMT in the 20% FEBSG group and the control group, based on prior findings from carcass performance and pork quality evaluation. Principal component analysis (PCA) revealed that 98.92% of the total variance was attributed to the PC1, with an additional 0.46% accounted for by the PC2 (Fig. 4A). The correlation analysis among samples indicated a high degree of correlation for samples within the same group (Fig. 4B). These findings suggest that the repeatability of samples within each group was satisfactory. A comparative analysis between the 20% FEBSG and Con groups revealed a total of 69 differentially expressed proteins. Among these, 51 proteins exhibited down-regulation, while 18 proteins showed up-regulation (Fig. 4C). Table S2A listed the up- and down-regulated proteins, and Fig. 4D showed the differential protein expression levels of the top 3 (up and down, respectively) with the biggest fold change values. The expressions of TNNC1, SDHA and TNNT3 were upregulated, while the expressions of HDAC4, BAG5 and USP5 were downregulated. The data highlighted significant disparities in protein expression characteristics between the 20% FEBSG and Con groups. GO analysis categorized the differentially expressed proteins into three main categories: biological processes (BP, with 104 identified proteins), molecular functions (MF, with 55 proteins), and cellular components (CC, with 20 proteins) (Table S2B). The top 10 enriched GO terms for each category—BP, CC, and MF—are graphically represented in Fig. 4E. In the analysis between the 20% FEBSG group to the Con group, several key biological processes were significantly affected, encompassing the negative regulation of transcription by RNA polymerase II (GO:0000122), regulation of muscle contraction (GO:0006937), and skeletal system development (GO:0001501). The primary molecular function classes impacted were glutathione peroxidase activity (GO:0004602), oxidoreductase activity (GO:0016628), ATPase activator activity (GO:0001671), and fatty acid binding (GO:0005504). Additionally, the principal cellular protein classes identified were mitochondrial respiratory chain complex I (GO:0005747), cytosol (GO:0005829), and cytoplasm (GO:0005737). Table S2C and Fig. 4F displayed the enriched pathways of differentially expressed proteins related to muscle growth based on KEGG analysis. Proteins that were differentially expressed in the 20% FEBSG group were predominantly associated with 40 distinct metabolic pathways. Notably, pathways related to the biosynthesis of amino acids (ssc01230), glutathione metabolism (ssc00480), AMPK signaling pathway (ssc04152), PPAR signaling pathway (ssc03320) and regulation of actin cytoskeleton (ssc04810) were enriched, indicating their crucial roles in the biological responses observed in this group.

### Metagenomics analysis of the colon

**Fig. 5:**
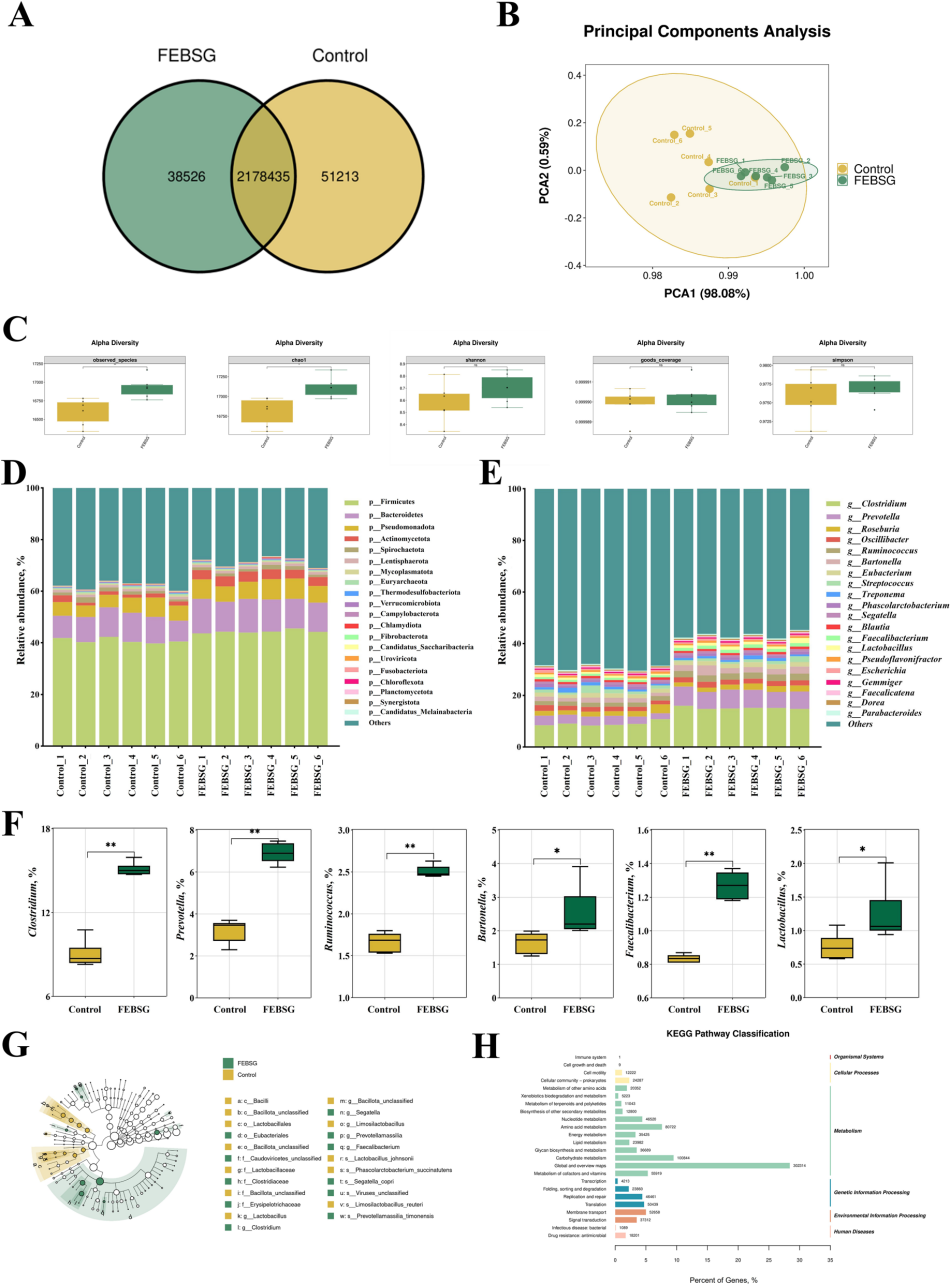
Metagenomics analysis of colon. **A** Venn plot showing the similarity and specificity of Unigenes between the FEBSG and control groups. **B** Principal coordinate analysis based on Bray–Curtis distance. **C** Alpha diversity for two groups of pigs. **D** Relative abundance at the bacterial phylum levels. **E** Relative abundance at the bacterial genus levels. **F** Average abundance of predominant genera in the groups. **G** Identification of crucial gut bacteria in the colon of two groups of pigs by LEfSe analysis. **H** Unigenes assigned to 45 level 2 KEGG items. ^*^P < 0.05, ^**^P < 0.01

A total of 2,268,174 high-quality Unigenes were obtained, with 2,178,435 Unigenes being present in both groups as a result of the whole-genome shotgun sequencing of 12 colon digesta samples. A significant increase in the count of Unigenes was observed in the 20% FEBSG group compared to the Con group, as depicted in Fig. 5A. Principal coordinates analysis (PCoA) further revealed that the bacterial community compositions between the two groups were significantly different, highlighting a distinct separation in their microbial signatures, as illustrated in Fig. 5B. Analysis of bacterial α-diversity revealed significant increases in both observed_species and Chao1 indices in the 20% FEBSG group when compared to the Con group (Fig. 5C). This suggests a richer bacterial diversity and greater bacterial abundance in the FEBSG group. We conducted an analysis of the microbiota composition in colon digesta samples at various taxonomic levels. Firmicutes predominated in the gut microbiome of both the Con and FEBSG group pigs at the phylum level, comprising 40.79% and 44.29% of the microbiota, respectively. Bacteroidetes followed, accounting for 9.94% in the Con group and 12.24% in the FEBSG group (Fig. 5D, Table S3A). At the genus level, *Clostridium* was notably abundant, representing 9.00% in the Con group and 15.10% in the FEBSG group. *Prevotella* was also prevalent, with 3.22% in the Con group and 6.91% in the FEBSG group (Fig. 5E, Table S3B). Furthermore, the colon digesta of the FEBSG group pigs exhibited a significant enrichment of *Clostridium*, *Prevotella*, *Bartonella*, *Ruminococcus*, *Faecalibacterium*, and *Lactobacillus* (Fig. 5F). Linear discriminant analysis (LEfSe) revealed elevated activity levels of bacterial genera such as *Clostridium*, *Prevotellamassilia* and *Faecalibacterium* in the FEBSG group, as illustrated in Fig. 5G. This indicated a potential shift in the microbial community composition in response to FEBSG supplementation. In the functional annotation analysis of the colon, Unigenes were classified into 24 level 2 KEGG pathways, encompassing six broad KEGG categories. Strikingly, a higher proportion of Unigenes in the colon digesta was linked to carbohydrate metabolism and amino acid metabolism, suggesting these metabolic pathways may be particularly active or influenced by the treatment, as shown in Fig. 5H. This highlighted the importance of these metabolic functions in the context of the study.

### Short-chain fatty acid analysis

**Fig. 6:**
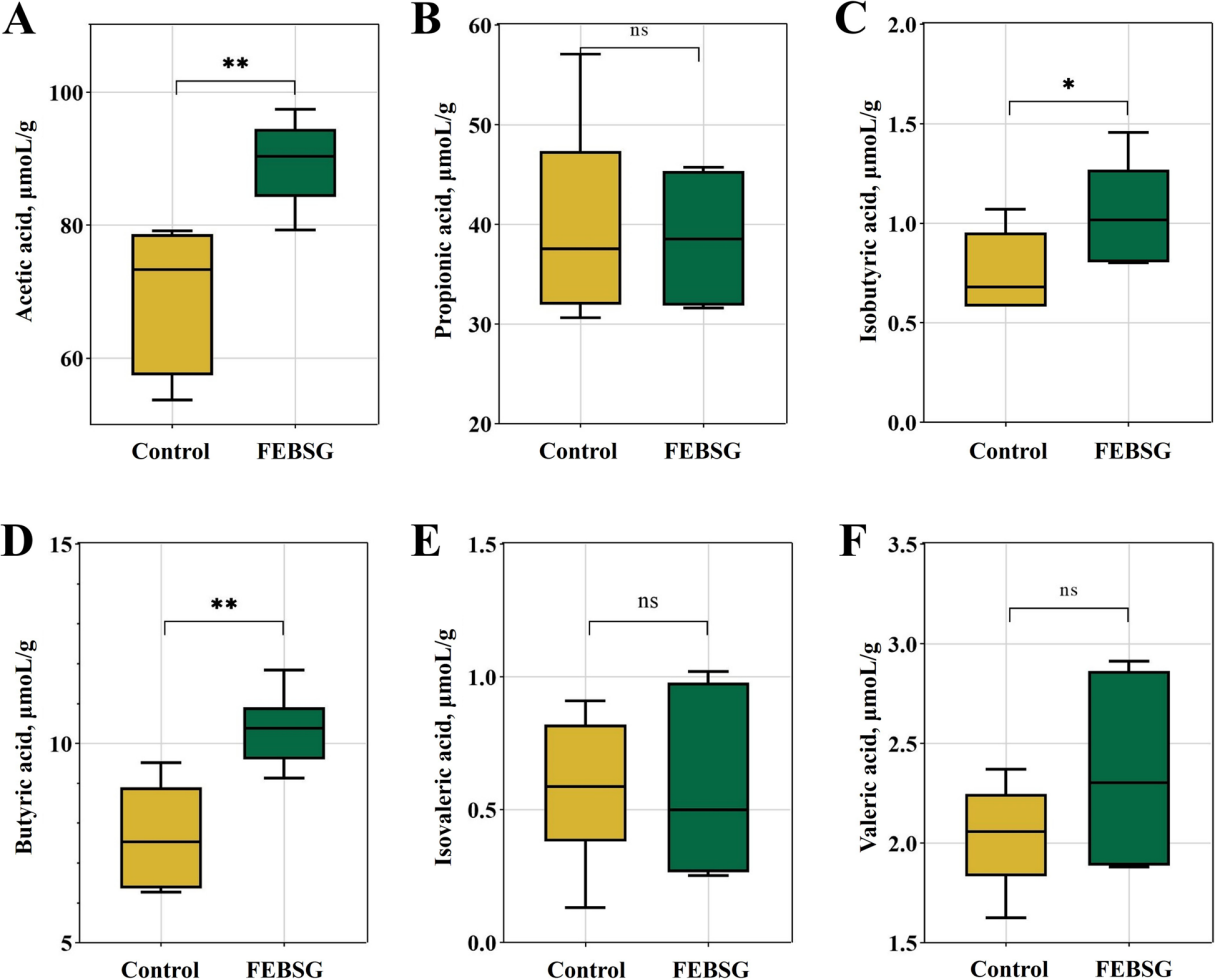
Effects of FEBSG treatment on the contents of volatile fatty acids in colon digesta of growing-finishing pigs. **A** Acetic acid. **B** Propionic acid. **C** Isobutyric acid. **D** Butyric acid. **E** Isovaleric acid. **F** Valeric acid. ^*^P < 0.05, ^**^P < 0.01

Figure 6 presented the levels of short-chain fatty acids in the colon. The findings indicated that the levels of AA, BA, and IBA in the colonic contents of pigs fed with diets containing 20% FEBSG were higher (*P* < 0.05) compared to those fed with the Con. Nonetheless, no notable differences (*P* > 0.05) were found in the levels of PA, VA, and IVA between the 20% FEBSG group and the Con group.

### Correlative analysis of proteomics and metagenomics data with meat quality characteristics

**Fig. 7:**
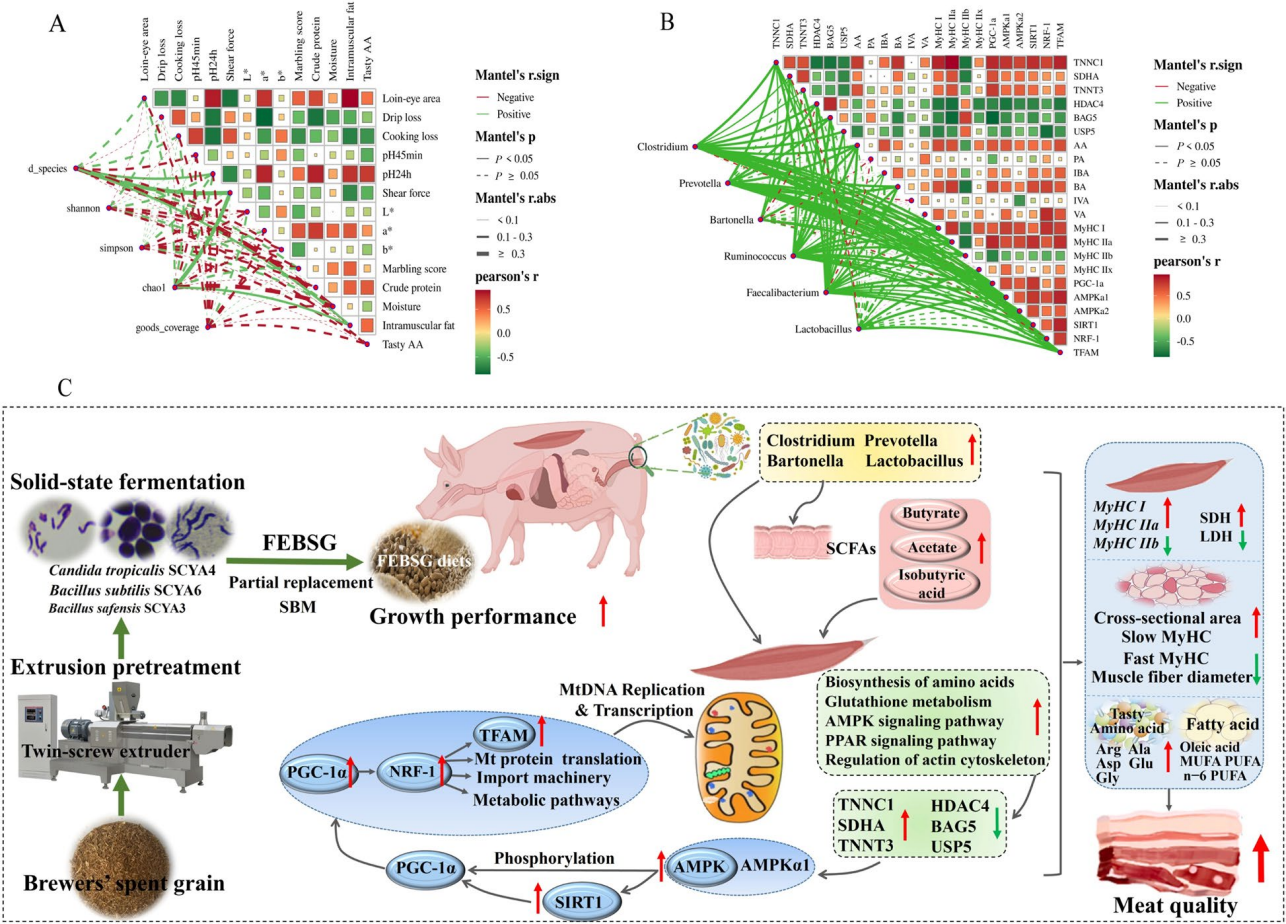
Correlation analysis between the proteomics and metagenomics data, and meat quality traits. **A** Correlation analysis between the alpha diversity of the bacteriome and meat quality traits in Control and FEBSG group. **B** Correlation analysis among the predominant genera, the differential expression protein, volatile fatty acids, muscle fiber characteristics and AMPK signal. **C** Integrated mechanism through which 20%FEBSG may improve skeletal muscle development and muscle fiber characteristics by shaping the gut microbiota and ultimately affect meat quality

To gain further insights into the impact and potential regulatory mechanisms of FEBSG on pig production, a correlation analysis was performed. The analysis focused on the relationships between the alpha diversity of the bacteriome and meat quality traits. Mantel’s test was employed to determine these correlations. As shown in Fig. 7A, pH_24h_, shear force, and intramuscular fat were found to be correlated with observed_species, with Mantel’s *r* values of 0.19, 0.52, and 0.25, respectively. Additionally, the shear force and intramuscular fat correlated with the Chao1 index. The loin-eye area showed a significant positive correlation with intramuscular fat (*r* = 0.91) and pH_24h_ (*r* = 0.83). Conversely, it was inversely related to cooking loss (*r* = −0.70) and shear force (*r* = −0.79). Drip loss and cooking loss were both negatively associated (*P* < 0.05) with intramuscular fat, pH_24h_, and the a* value. Additionally, pH_24h_ demonstrated a positive (*P* < 0.05) correlation with intramuscular fat, tasty AA, crude protein, and the a* value (*r* = 0.75, *r* = 0.71, and *r* = 0.82, respectively). pH_24h_ also correlated inversely with shear force (*r* = −0.69). Figure 7B presented a correlation analysis among predominant genera, differentially expressed proteins, volatile fatty acids, muscle fiber types, and AMPK signaling characteristics. The analysis showed significant correlations (*P* < 0.05) between TNNC1 and *AMPKα1* with *Clostridium*, *Prevotella*, *Bartonella*, *Ruminococcus*, *Faecalibacterium*, and *Lactobacillus*. Additionally, SDHA showed correlations (*P* < 0.05) with *Clostridium* (*r* = 0.26), *Bartonella* (*r* = 0.47), *Ruminococcus* (*r* = 0.23), and *Faecalibacterium* (*r* = 0.24). TNNT3 was significantly related with *Clostridium* and *Faecalibacterium* (*P* < 0.05). Furthermore, HDAC4, AA, BA, *MyHC I*, *MyHC IIa*, *AMPKα1*, *MyHC IIb*, *SIRT1*, *PGC-1a*, *NRF-1* and *TFAM* were each correlated with *Clostridium*, *Prevotella*, *Ruminococcus*, and *Faecalibacterium* (*P* < 0.05). TNNC1, SDHA, and TNNT3 were positively associated with *MyHC IIa* and *PGC-1a*, and negatively associated with *MyHC IIb* and USP5 (*P* < 0.05). HDAC4 was negatively associated with *MyHC I*, *MyHC IIa*, *AMPKα1*, *SIRT1*, and *TFAM*, while showing a positive correlation with BAG5 (*P* < 0.05). BA demonstrated positive correlations with TNNC1, *MyHC IIa*, *PGC-1a*, *SIRT1*, and *NRF-1*, and negative correlations (*P* < 0.05) with BAG5 and *MyHC IIb*. *MyHC I* and *MyHC IIa* were positively correlated with *TFAM*, *AMPKα1*, *AMPKα2*, *SIRT1*, and *NRF-1*, and negatively correlated with *MyHC IIb* (*P* < 0.05). We propose that 20% FEBSG may enhance muscle fiber characteristics by modulating the gut microbiota, thereby improving meat quality (Fig. 7C).

## Discussion

In this study, we observed that substituting SBM with FEBSG in the diets of finishing pigs resulted in an increase in final body weight, average daily gain, average daily feed intake, carcass weight and loin-eye area. These results indicated that FEBSG can positively influence growth performance and carcass characteristics in pigs, corroborating earlier finding [8]. Prior investigations have established that the use of microbial fermented feed can confer multiple advantages for animal growth, including increased feed consumption, enhanced nutrient absorption, and improved intestinal health. These beneficial effects are largely due to the microorganisms’ capacity to break down anti-nutritional factors and complex nutrients in the feed, as reported by Su et al. [24].

Pork quality encompasses both sensory and nutritional aspects. The assessment of sensory quality in pork primarily focuses on attributes including meat color, drip loss, taste, pH, marbling, tenderness, and juiciness [25]. These indicators are crucial factors influencing consumers' purchasing decisions. Notably, meat color stands out as a primary quality attribute of meat products, directly shaping consumer preferences. Within a certain range, pork pH was positively correlated with meat color scores [26]. While the pH affects meat color, tenderness and drip loss [27]. The nutritional quality of pork primarily pertains to its nutritional value, encompassing the levels of AA, fatty acids, minerals, and other nutrients. This quality is closely linked to human nutrition and health [28]. Notably, the AA levels in pork are positively correlated with the meat’s taste and nutritional value [29]. The nutritional value of pork can also be assessed by analyzing the fatty acid profile. Within this profile, the level of saturated fatty acids is typically inversely related to the perceived flavor of the meat, while the concentration of unsaturated fatty acids correlates positively with a more desirable meat taste [30]. It is important to highlight that unsaturated fatty acids, particularly oleic acid (C18:1n9c), have been recognized for their potential to help prevent cardiovascular diseases [31]. Human dietary standards specifically recommend consuming more unsaturated fatty acids and reducing foods with high saturated fatty acids content [32], which may help to select better quality pork. Consistent with prior research [33, 34], this study found that 20% FEBSG group had enhanced meat color and marbling, along with higher levels of tasty AA and monounsaturated fatty acids in the LT than the Con group. Moreover, there was a notable decrease in shear force. The improvement in meat quality is likely due to the probiotic strains present in the microbial fermented feed, as their bioactive metabolites are known to enhance pork quality positively [8]. These results support the notion that supplementing diets with FEBSG can elevate pork quality, with particularly notable effects in the 20% FEBSG group.

Actually, pork quality is the outcome of the extended development and metabolic processes of muscles. Muscle fiber attributes, including their quantity, size, fiber density, and type, are indicative of muscle development and metabolic status, and they significantly influence muscle quality. Among them, the muscle fiber density and diameter are key determinants of pork’s water retention, tenderness, and losses during drip and cooking processes. The smaller the diameter of muscle fibers, the more intramuscular fat, the more tender the meat, the less the dripping loss and cooking loss [35]. In terms of muscle fiber types, there are four main types: MyHC I (slow oxidative), MyHC IIa (fast oxidative), MyHC IIx (intermediate) and MyHC IIb (fast glycolytic) [36]. Research indicates that a greater levels of MyHC IIb is associated with increased lightness, rapid and substantial pH drop after slaughter, but decreased water-holding capacity [37]. Conversely, a predominance of MyHC I and MyHC IIa fibers is associated with greater redness, higher postmortem pH, and improved water retention in meat [38]. Moreover, type I fibers also contributed to meat tenderness, primarily due to their reduced fiber diameter [39]. This research revealed that FEBSG notably reduced the muscle fiber diameter and the expression levels of *MyHC IIb*, while enhancing the CSA and expression of *MyHC I* and *MyHC IIa*. Immunofluorescence analysis confirmed these trends in both fast and slow muscle fibers within the LT. Consequently, the enhancements in meat color and the reductions in drip loss, cooking loss, shear force, and muscle fiber diameter in the FEBSG group are likely attributed to the upregulation of *MyHC I* and *MyHC IIa*. Moreover, the activity of enzymes associated with muscle energy metabolism, including SDH, MDH, and LDH, have an indirect effect on the distribution of muscle fiber types. Specifically, elevated SDH activities are correlated with a larger presence of slow-twitch muscle fibers, whereas higher LDH activity correlates with a greater proportion of fast-twitch fibers [40]. In our study, the FEBSG-treated group exhibited higher SDH activity and lower LDH activity in the LT. These findings suggest that incorporating FEBSG in place of SBM in the diet can induce a transformation in muscle fiber types, consequently enhancing the pork quality of pigs.

Wang et al. demonstrated that muscle fiber types are intimately connected to mitochondrial content, and the biogenesis of mitochondria can promote the shift from fast-twitch to slow-twitch muscle fibers [41]. The AMPK pathway plays a crucial role in this process, as AMPK can phosphorylate *PGC-1α*, activate related signaling, regulate *NRFs*, and enhance *TFAM* expression, thus driving mitochondrial biogenesis and the development of slow muscle fibers [42]. In our research, we noticed that FEBSG upregulated the expression of key genes in the AMPK/PGC-1α pathway, including *AMPKα1*, *PGC-1α*, *NRF1*, and *TFAM*. This suggests that FEBSG activates the AMPK/PGC-1α/TFAM signal, potentially explaining the observed shift towards slow muscle fibers in pigs.

Utilizing TMT-based quantitative proteomics, we investigated the potential molecular mechanism of regulating pork quality. In our research, 18 proteins were found to be upregulated and 51 downregulated in the FEBSG group compared to the Con group. Notably, FEBSG upregulated TNNC1 (Troponin C), TNNT3 (Troponin T), and Succinate dehydrogenase (SDHA), while downregulating histone deacetylase 4 (HDAC4). TNNC1 and TNNT3 are suggested to be potential markers for meat color and tenderness [43, 44], which could explain the improved pork quality observed with FEBSG treatment. SDHA, a crucial subunit of succinate dehydrogenase, is significant for mitochondrial function [45]. HDAC4, a class IIa histone deacetylase, is known to regulate PGC-1α, a key factor in muscle metabolism and mitochondrial biogenesis [46]. We speculated that FEBSG enhances meat quality by modulating the expression of these proteins. Additionally, our functional annotation and pathway analyses revealed that these proteins with altered expression were notably concentrated in specific pathways like the AMPK signaling pathway, amino acid biosynthesis, glutathione metabolism, PPAR signaling pathway, and actin cytoskeleton regulation, these signaling pathways regulate the meat quality through various potential mechanisms [47–49].

Recent research has highlighted the link between the intestinal microbiota and muscle development, with the "gut-muscle axis" emerging as a key area of interest. This theory suggests that gut microbiota can affect muscle phenotype [47]. We speculate that FEBSG might influence muscle phenotype by modulating this gut-muscle axis. Metagenomics sequencing was employed to compare microbial populations in the colon contents of the two study groups, revealing significant enrichment of *Clostridium*, *Prevotella*, *Bartonella* and *Lactobacillus* in the FEBSG group. *Clostridium* is known to impact amino acid metabolism and muscle development [50], while *Lactobacillus*, with its anti-inflammatory effects, is essential for muscle mass maintenance [51]. Fielding et al. noted that individuals with normal muscle function exhibit greater quantities of *Prevotella* and *Bartonella* in their intestinal microbiota [52]. Additionally, *Barnesiella* and *Prevotella* are known short-chain fatty acid producers [53], providing a plausible explanation for the considerable rise in the levels of AA, BA and IBA in the colonic contents of pigs fed a 20% FEBSG diet in this research. It is noteworthy that short-chain fatty acids can phosphorylate *AMPK*, and activated *AMPK* can phosphorylate the downstream target *PGC-1α* and can stimulate *SIRT1*, activating *PGC-1α* [42]. As previously stated, PGC-1α is pivotal in the regulation of mitochondrial function, biogenesis, and the development of slow-twitch muscle fibers. The AMPK/PGC-1α/TFAM signaling pathway was found to be activated in our study. This once again illustrates that the gut microbiota and its metabolic products, short-chain fatty acids, may serve as potential signaling molecules, participating in the modulation of muscle development and metabolism through the gut-muscle axis, and ultimately regulating pork quality [20]. However, the relationships among gut microbiota, metabolites, and muscle signaling pathways remain to be further validated.

## Conclusions

Our findings demonstrate that partial replacement of soybean meal with FEBSG in growing-finishing pigs improves growth performance, carcass traits, and meat quality. Based on an overall evaluation of growth performance, carcass characteristics, and meat quality, 20% FEBSG was identified as the optimal dietary inclusion level for growing–finishing pigs. Mechanistically, dietary supplementation with FEBSG enhances pork quality by activating the AMPK/PGC-1α/TFAM signaling pathway in muscle via the gut-muscle axis, thereby promoting mitochondrial biogenesis, muscle development, muscle fiber type transformation, and muscle metabolism. This study provides important evidence for understanding the mechanisms by which probiotic-fermented agricultural byproducts improve pork quality, and the feed formulation proposed by the study also offers new ideas for alleviating the pressure of competition for food resources between humans and animals, as well as the tension in soybean meal resources.

## Supplementary Information

Additional file 1: Table S1. Primer sequences used in this study. 

Additional file 2: Table S2A. Differential proteins. Table S2B. GO annotation result of differentially expressed proteins. Table S2C. KEGG pathway enrichment analysis.

Additional file 3: Table S3A. Relative abundance at the bacterial phylum levels. Table S3B. Relative abundance at the bacterial genus levels.

## Data Availability

Data will be provided upon request.

## Abbreviations

AA: Amino acid
ADFI: Average daily feed intake
ADG: Average daily gain
AMPK: AMP-activated protein kinase
BW: Body weight
FEBSG: Fermented extruded brewers’ spent grain
F/G: Feed to gain ratio
LT: Longissimus thoracis
MyHC: Myosin heavy chain
MUFA: Total monounsaturated fatty acid
NRF: Nuclear respiratory factor
PGC-1α: Peroxisome proliferator activated receptor-gamma coactivator-1alpha
PUFA: Polyunsaturated fatty acid
SFA: Saturated fatty acid
SIRT1: Sirtuin 1
TFAM: Mitochondrial transcription factor A
