# Supplementary material for: Integrated gut metagenomic and muscle proteomic analysis reveals the role of dietary fermented extruded brewers’ spent grain in enhancing pork quality through the gut-muscle axis
Source: J Anim Sci Biotechnol. 2026 Jun 6;17:111. doi: 10.1186/s40104-026-01429-4 (PMC13242122; doi:10.1186/s40104-026-01429-4)
Supplement: Supplementary file 1 — Additional file 1: Table S1. Primer sequences used in this study. [file 40104_2026_1429_MOESM1_ESM.docx]

**Appendix A. Supplementary data**

**The following are the Supplementary data to this article:**

**Table S1 Primer sequences used in this study**

| Gene name | Primers | Sequences, 5’-3’ | Product Size, bp |
| --- | --- | --- | --- |
| *MyHC I* | Forward | CGTGGACTACAACATCATAGGC | 146 |
|  | Reverse | CCTTCTCAACAGGTGTGTCG |  |
| *MyHC IIa* | Forward | CATTGAGGCCCAGAATAGGC | 127 |
|  | Reverse | TGCTTCCGTCTTCACTGTCAC |  |
| *MyHC IIb* | Forward | GACTCTGGCTTTCCTCTTTGC | 101 |
|  | Reverse | GAGCTGACACGGTCTGGAAA |  |
| *MyHC IIx* | Forward | TTGACTGGGCTGCCATCAAT | 111 |
|  | Reverse | GCCTCAATGCGCTCCTTTTC |  |
| *PGC-1a* | Forward | CCAGTACAACAATGAGCCTGC | 118 |
|  | Reverse | CAATCCGTCTTCATCCACG |  |
| *AMPKa1* | Forward | CGGCAAAGTGAAGGTTGG | 123 |
|  | Reverse | AGGTTCTGAATTTCTCTGCGG |  |
| *AMPKa2* | Forward | GGAGGTTCTCAGCTGCCTTT | 132 |
|  | Reverse | GAATCAGGTGGGCTTGTTGC |  |
| *TFAM* | Forward | GCTCTCCGTTCAGTTTTGCG | 187 |
|  | Reverse | GGAAGTTCCCTCCACAGCTC |  |
| *SIRT1* | Forward | ACTCTCCCTCTTTTAGACCAAGC | 149 |
|  | Reverse | AAACCTGGACTCTCCATCGG |  |
| *NRF-1* | Forward | CCTTGTGGTGGGAGGAATGTT | 77 |
|  | Reverse | AGTATGCTGGCTGACCTTGTG |  |
| *GADPH* | Forward | ACTCACTCTTCTACCTTTGATGCT | 100 |
|  | Reverse | TGTTGCTGTAGCCAAATTCA |  |

MyHC= myosin heavy chain; PGC-1𝛼=peroxisome proliferator activated receptor-gamma coactivator (PGC)-1alpha; AMPK=AMP-activated protein kinase; TFAM=mitochondrial transcription factor A; SIRT1=sirtuin 1; NRF=nuclear respiratory factor.
